# Delayed Ovarian Teratoma Resection in Anti–N-Methyl-D-Aspartate (NMDA) Receptor Encephalitis Despite Immunotherapy: Implications for Early Source Control

**DOI:** 10.7759/cureus.116224

**Published:** 2026-09-14

**Authors:** Jordan Scott, Brittany L Lawrence, Khalid Mehmood

**Affiliations:** 1 Osteopathy, Liberty University College of Osteopathic Medicine, Lynchburg, USA; 2 Neurology, Liberty University College of Osteopathic Medicine, Lynchburg, USA; 3 Internal Medicine, Chesapeake Regional, Chesapeake, USA

**Keywords:** anti-nmda receptor encephalitis, autoimmune encephalitis, early tumor removal, immunotherapy, ovarian teratoma

## Abstract

Anti-N-methyl-D-aspartate (NMDA) receptor encephalitis is a severe autoimmune neurologic disorder frequently associated with ovarian teratomas in adult women. Tumor removal is a central component of management and is associated with improved neurologic outcomes. We report a 33-year-old woman with confirmed anti-NMDA receptor encephalitis complicated by refractory seizures, movement disorder, and prolonged ventilator dependence in whom ovarian teratoma resection was delayed after the family initially declined surgical intervention.

Prior to surgical intervention, the patient received high-dose corticosteroids, plasma exchange (PLEX), intravenous immunoglobulin (IVIG), and rituximab, without meaningful clinical improvement. Pelvic imaging repeatedly failed to identify a definitive ovarian teratoma; however, gynecologic consultation recommended bilateral salpingo-oophorectomy. During the interval between the initial surgical recommendation and eventual family consent, the patient continued to demonstrate persistent severe neurologic dysfunction despite ongoing supportive and immunomodulatory therapy. Bilateral salpingo-oophorectomy was ultimately performed, and surgical pathology confirmed a mature ovarian teratoma. Despite tumor removal and escalation to cyclophosphamide, the patient remained ventilator-dependent with profound neurologic impairment.

This case highlights the potential limitations of immunotherapy when an underlying antigenic source remains present and emphasizes the importance of maintaining suspicion for an occult ovarian teratoma and considering timely tumor-directed therapy in severe anti-NMDA receptor encephalitis despite inconclusive imaging.

## Introduction

Anti-N-methyl-D-aspartate (NMDA) receptor encephalitis is an autoimmune encephalitis mediated by IgG antibodies against the glutamate ionotropic receptor NMDA type subunit 1 (GluN1), resulting in receptor internalization and synaptic dysfunction [1,2]. The disorder often begins with acute psychiatric symptoms followed by seizures, movement disorders, autonomic instability, and decreased level of consciousness [1,3,4]. In women older than 18 years, approximately 50-60% of cases are associated with ovarian teratomas [1,5]. Ovarian teratomas may contain neural tissue expressing NMDA receptors, which can serve as an antigenic stimulus for the autoimmune response [1,3].

Removal of an identified teratoma is considered to be a cornerstone of therapy and is associated with improved functional outcomes and lower relapse rates [5,6]. Immunotherapy commonly includes corticosteroids, intravenous immunoglobulin (IVIG), plasma exchange (PLEX), and second-line agents such as rituximab and cyclophosphamide [5,6]. Small ovarian teratomas may be occult on pelvic imaging, and the persistence of an antigenic source may sustain the autoimmune response [2,7]. This case illustrates the clinical implications of delayed tumor removal despite aggressive immunotherapy in severe treatment-refractory anti-NMDA receptor encephalitis with nondiagnostic imaging.

## Case presentation

A 33-year-old woman with a history of asthma, obstructive sleep apnea, and depression initially developed headache, fatigue, myalgias, and hypersomnia. Over the following days, she developed agitation, confusion, and erratic behavior, followed by multiple generalized seizures progressing to status epilepticus. Her neurologic condition subsequently deteriorated with progressive encephalopathy, abnormal movements, respiratory failure, and impaired airway protection, ultimately requiring tracheostomy and percutaneous endoscopic gastrostomy placement.

Cerebrospinal fluid (CSF) analysis demonstrated an elevated opening pressure, mild lymphocytic pleocytosis, positive oligoclonal bands, an elevated IgG index, and anti-NMDA receptor antibodies at a titer of 1:320, confirming anti-NMDA receptor encephalitis. The relevant cerebrospinal fluid (CSF) findings are summarized in Table 1.

**Table 1:** Cerebrospinal fluid findings supporting the diagnosis of anti-NMDA receptor encephalitis Cerebrospinal fluid analysis demonstrated elevated opening pressure, mild lymphocytic pleocytosis, positive oligoclonal bands, elevated IgG index, and anti-NMDA receptor antibodies, supporting the diagnosis of anti-NMDA receptor encephalitis. WBC: white blood cell count; IgG: immunoglobulin G; NMDA: N-methyl-D-aspartate; CSF: cerebrospinal fluid

Test	Observed Value	Reference Range/Interpretation
Opening Pressure	49 cm H2O	10-25 cm H2O
WBC	20 cells/μL	0-5 cells/μL
Lymphocytes	97%	Lymphocytic Predominance
Protein	54 mg/dL	15-45 mg/dL
Glucose	82 mg/dL	Preserved CSF Glucose
Oligoclonal Bands	10	Positive
IgG Index	1.6	<0.7
NMDA Antibody Titer	1:320	Positive

Brain magnetic resonance imaging (MRI) demonstrated mild diffuse cortical fluid-attenuated inversion recovery (FLAIR) signal abnormalities without focal lesions. Continuous electroencephalographic monitoring later demonstrated severe diffuse encephalopathy with extreme delta brush; no electrographic seizures or epileptiform discharges were identified during this monitoring period. Clinically, she developed persistent orofacial dyskinesias, chorea, dystonia, autonomic instability, and ventilator dependence.

High-dose intravenous methylprednisolone was administered for five days, followed by five sessions of PLEX. Because of persistent severe neurologic dysfunction, rituximab was subsequently initiated with two initial doses administered two weeks apart, followed by planned monthly dosing. Despite corticosteroids, PLEX, and rituximab, she remained critically ill with persistent neurologic dysfunction.

Serial evaluation for an underlying ovarian teratoma was pursued because of the association between ovarian teratomas and anti-NMDA receptor encephalitis. Three pelvic MRI examinations failed to demonstrate a definitive ovarian teratoma, identifying only a small left ovarian hemorrhagic follicle or cyst. A transvaginal ultrasound was also obtained during the evaluation (Figure 1).

**Figure 1:**
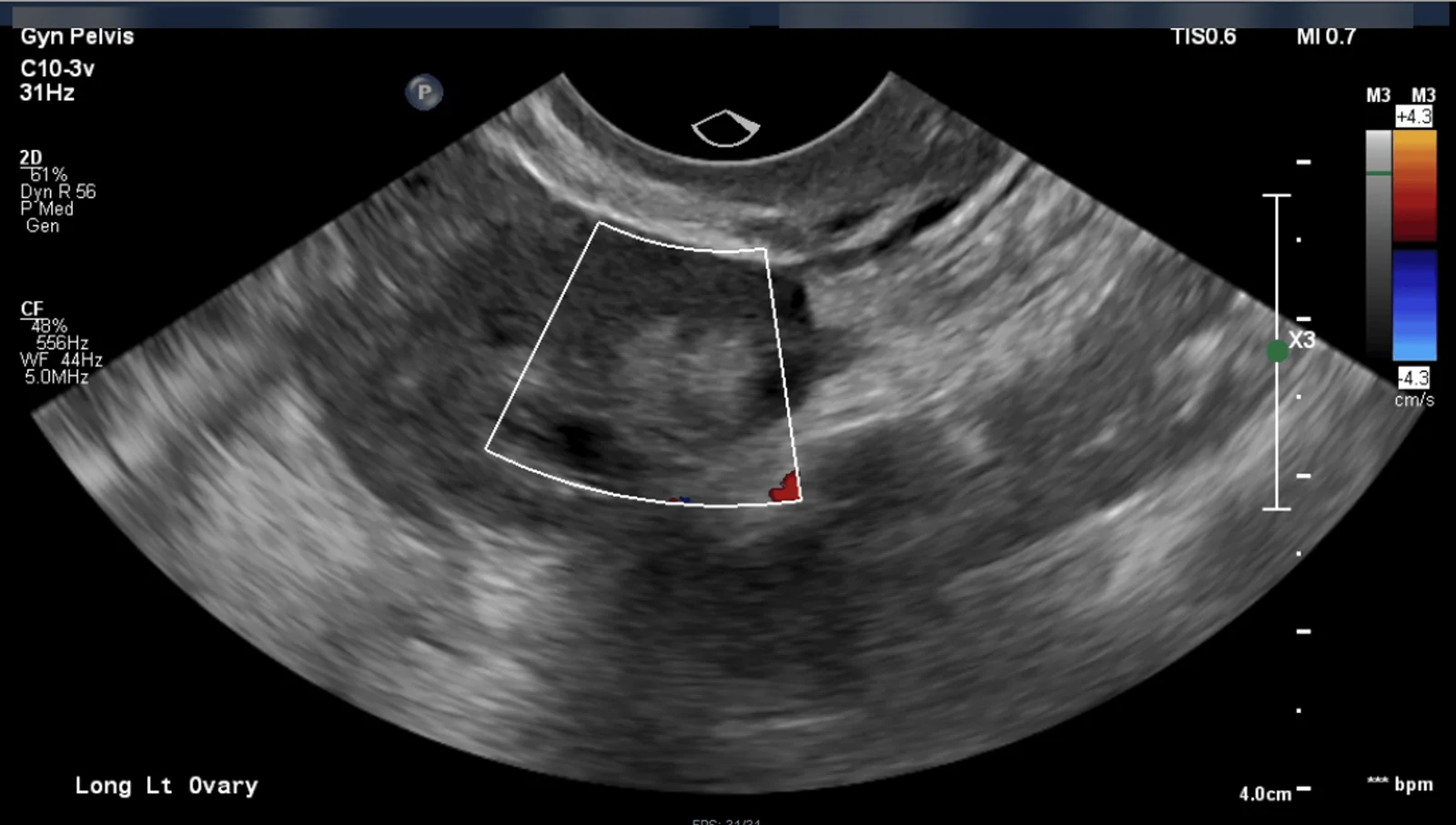
Transvaginal ultrasound obtained during evaluation for an occult ovarian teratoma Transvaginal ultrasound of the left ovary obtained during the evaluation for a suspected ovarian teratoma in a patient with anti-NMDA receptor encephalitis. Subsequent pelvic MRI examinations did not demonstrate a definitive teratoma; surgical pathology following bilateral salpingo-oophorectomy ultimately confirmed a mature ovarian teratoma.

Given the patient's refractory disease and persistent concern for an occult ovarian teratoma, gynecology recommended bilateral salpingo-oophorectomy (BSO) despite the absence of a definitive teratoma on pelvic MRI. The patient's family initially declined surgical intervention despite multidisciplinary counseling. Twelve days later, following continued discussion, consent was obtained, and BSO was performed. Surgical pathology subsequently confirmed a mature ovarian teratoma.

Following tumor removal, the serum anti-NMDA receptor antibody titer decreased from 1:2560 before surgery to 1:640. Despite this decrease in antibody titer, there was no corresponding meaningful neurologic improvement. However, severe neurologic dysfunction persisted, including dyskinesias, paroxysmal sympathetic hyperactivity, and ventilator dependence. Immunotherapy was escalated to cyclophosphamide, followed by additional intravenous immunoglobulin and repeat PLEX because of persistent disease.

The major diagnostic and therapeutic events during the prolonged hospital course are summarized in Table 2.

**Table 2:** Major events in the clinical course and management of anti-NMDA receptor encephalitis Condensed timeline from symptom onset through treatment escalation, bilateral salpingo-oophorectomy, pathologic confirmation of ovarian teratoma, and transition to comfort-focused care. IV: intravenous; PEG: percutaneous endoscopic gastrostomy; CSF: cerebrospinal fluid; NMDA: N-methyl-D-aspartate; MRI: magnetic resonance imaging; PLEX: plasma exchange; EEG: electroencephalography; IVIG: intravenous immunoglobulin; BSO: bilateral salpingo-oophorectomy

Day	Major event
0	Headache, fatigue, myalgias, and hypersomnia begins.
3-7	Repeated emergency evaluations are followed by agitation, confusion, erratic behavior, generalized seizures, status epilepticus, and hospital admission.
20-24	IV methylprednisolone is started for five days. Tracheostomy and PEG are placed.
32	CSF anti-NMDA receptor antibody titer is 1:320. Pelvic MRI #1 shows no teratoma and a probable small left hemorrhagic follicle.
34-40	PLEX is administered for five sessions.
37	Transfer to tertiary neurologic intensive care for advanced management.
41-55	Serum antibody titer rises to 1:1280. Transfer to second tertiary neurological center for enrollment in ExTINGUISH clinical trial; not a candidate. Pelvic MRI #2 and #3 remain negative for teratoma; brain MRI shows mild diffuse cortical abnormalities; continuous EEG demonstrates extreme delta brush. Initial IVIG is started for five doses; discontinued after fourth dose due to excess urine output. Rituximab is started.
66-76	Prednisone taper. Transfer back to the initial referring tertiary hospital. Gynecology recommends BSO on day 76 despite nondiagnostic pelvic MRI. Maintenance IVIG is started, once weekly doses for six weeks.
77-78	Serum antibody titer is 1:2560. Family declines surgery on day 78.
90	Family agrees to surgery; bilateral salpingo-oophorectomy is performed.
99	Surgical pathology confirms a mature ovarian teratoma.
101	Serum antibody titer decreases to 1:640. Cyclophosphamide is initiated.
112-183	Maintenance IVIG is administered every two weeks for six weeks. Repeat PLEX is administered for five sessions for persistent disease. Transferred back to referring community hospital neurological intensive care unit.
237	Planned rituximab course is completed.
264-274	Sedation is reduced as the family considers comfort-focused care. Discharge to comfort care occurs on day 272. Death occurs on day 274.

Her prolonged intensive care course was complicated by severe tongue trauma with macroglossia secondary to recurrent tongue biting, nosocomial infections, venous thrombosis, and renal injury. Despite extensive immunotherapy, tumor removal, and prolonged supportive care, neurologic recovery remained limited. After nearly nine months of hospitalization, discussions with the family ultimately led to transition toward comfort-focused care.

## Discussion

This case highlights several important challenges in the management of anti-NMDA receptor encephalitis, particularly the role of tumor-directed therapy and the limitations of imaging in detecting ovarian teratomas. Despite aggressive immunotherapy, the patient experienced persistent severe neurologic dysfunction while an underlying antigenic source remained present. Surgical pathology ultimately confirmed a mature ovarian teratoma despite three pelvic MRI examinations that failed to identify a definitive lesion.

Ovarian teratomas contain ectopic neural tissue capable of expressing NMDA receptor antigens, which may sustain antibody production and perpetuate the autoimmune response [2,7,8]. Tumor removal therefore represents a key component of treatment rather than simply an adjunct to immunotherapy [5,6]. Observational data have demonstrated improved outcomes among patients receiving early treatment and tumor removal when a tumor is present [3,5]. In this patient, persistent severe disease despite corticosteroids, plasma exchange, and rituximab prompted continued concern for an occult teratoma despite unrevealing imaging.

Failure to detect an ovarian teratoma on imaging has been reported in patients with anti-NMDA receptor encephalitis, particularly when tumors are small or difficult to distinguish from physiologic ovarian findings [7]. In this case, three pelvic MRI examinations failed to demonstrate a definitive teratoma, identifying only a small left ovarian hemorrhagic follicle or cyst. Pathologic confirmation of a mature teratoma following bilateral salpingo-oophorectomy demonstrates the potential limitations of imaging and supports maintaining clinical suspicion for an occult teratoma in patients with severe or refractory disease.

Another important feature of this case was the delay between the recommendation for surgical intervention and eventual family consent. During this interval, the patient remained critically ill despite multiple immunomodulatory therapies. Although the effect of this 12-day delay on the patient's ultimate neurologic outcome cannot be established from a single case, it illustrates the importance of early multidisciplinary counseling regarding the therapeutic role of tumor removal when an occult teratoma is strongly suspected.

Neurologic recovery from anti-NMDA receptor encephalitis may be prolonged, particularly in patients with severe disease requiring intensive care support [1,5]. Despite tumor removal and subsequent escalation of immunotherapy with cyclophosphamide, IVIG, and repeat PLEX, this patient remained ventilator-dependent with severe dyskinesias and autonomic instability. After a prolonged hospitalization with limited neurologic recovery, care was ultimately transitioned toward comfort-focused measures. The patient’s course demonstrates that removal of a suspected antigenic source is an important component of management when a teratoma is present, but it does not guarantee rapid or complete neurologic recovery once disease severity progresses.

This case emphasizes the importance of maintaining suspicion for an occult ovarian teratoma in women with anti-NMDA receptor encephalitis despite inconclusive pelvic imaging. In patients with severe or treatment-refractory disease, multidisciplinary discussion regarding tumor-directed management may be warranted when clinical suspicion remains high.

## Conclusions

This case highlights the importance of timely tumor-directed therapy in anti-NMDA receptor encephalitis. Despite multiple unrevealing pelvic imaging studies, surgical pathology confirmed a mature ovarian teratoma, emphasizing the limitations of imaging and the need to maintain suspicion for an occult tumor in severe or refractory disease. In this patient, extensive immunotherapy did not result in meaningful neurological recovery while the underlying ovarian teratoma remained in situ. Multidisciplinary counseling regarding tumor removal should therefore be considered when clinical suspicion remains high despite inconclusive imaging.
